# Locality-preserving minimal perfect hashing of *k*-mers

**DOI:** 10.1093/bioinformatics/btad219

**Published:** 2023-06-30

**Authors:** Giulio Ermanno Pibiri, Yoshihiro Shibuya, Antoine Limasset

**Affiliations:** Ca’ Foscari University of Venice, Venice 30172, Italy; ISTI-CNR, Pisa 56124, Italy; University Gustave Eiffel, Marne-la-Vallée 77454, France; University of Lille, CRIStAL CNRS, UMR 9189 , Lille F-59000, France

## Abstract

**Motivation:**

Minimal perfect hashing is the problem of mapping a static set of *n* distinct keys into the address space {1,…,n} bijectively. It is well-known that $n{\;\text{log}\;}_{2}(e)$ bits are necessary to specify a minimal perfect hash function (MPHF) *f*, when no additional knowledge of the input keys is to be used. However, it is often the case in practice that the input keys have intrinsic relationships that we can exploit to lower the bit complexity of *f*. For example, consider a string and the set of all its distinct *k*-mers as input keys: since two consecutive *k*-mers share an overlap of k−1 symbols, it seems possible to beat the classic ${\;\text{log}\;}_{2}(e)$ bits/key barrier in this case. Moreover, we would like *f* to map consecutive *k*-mers to consecutive addresses, as to also preserve as much as possible their relationship in the codomain. This is a useful feature in practice as it guarantees a certain degree of locality of reference for *f*, resulting in a better evaluation time when querying consecutive *k*-mers.

**Results:**

Motivated by these premises, we initiate the study of a new type of locality-preserving MPHF designed for *k*-mers extracted consecutively from a collection of strings. We design a construction whose space usage decreases for growing *k* and discuss experiments with a practical implementation of the method: in practice, the functions built with our method can be several times smaller and even faster to query than the most efficient MPHFs in the literature.

## 1 Introduction

Given a universe set *U*, a function f:U→[n]={1,…,n} is a minimal perfect hash function (MPHF, henceforth) for a set S⊆U with n=|S| if f(x)≠f(y) for all x,y∈S, x≠y. In simpler words, *f* maps each key of *S* into a distinct integer in *[n]*. The function is allowed to return any value in *[n]* for a key x∈U∖S. A classic result established that $n{\;\text{log}\;}_{2}(e)=1.442n$ bits are essentially necessary to represent such functions for |U|≫n (Mehlhorn 1982). Minimal perfect hashing is a central problem in data structure design and has received considerable attention, both in theory and practice. In fact, many practical constructions have been proposed (see e.g. Pibiri and Trani 2021a and references therein). These algorithms find MPHFs that take space close to the theoretic-minimum, e.g. 2–3 bits/key, retain very fast lookup time, and scale well to very large sets. Applications of minimal perfect hashing range from computer networks (Lu et al. 2006) to databases (Chang and Lin 2005), as well as language models (Pibiri and Venturini 2019; Strimel et al. 2020), compilers, and operating systems. MPHFs have been also used recently in Bioinformatics to implement fast and compact dictionaries for fixed-length DNA strings (Almodaresi et al. 2018; Marchet et al. 2021; Pibiri 2022a,b).

In its simplicity and versatility, the minimal perfect hashing problem does not take into account specific types of inputs, nor the intrinsic relationships between the input keys. Each key x∈S is considered independently from any other key in the set and, as such, $P[f(x)=i]\approx \frac{1}{n}$ for any fixed i∈[n]. In practice, however, the input keys often present some regularities that we could exploit to let *f* act less randomly on *S*. This, in turn, would permit to achieve a lower space complexity for *f*.

We therefore consider in this article the following special setting of the minimal perfect hashing problem: the elements of *S* are all the distinct sub-strings of length *k*, for some k>0, from a given collection X of strings. The elements of *S* are called *k*-mers. The crucial point is that any two consecutive *k*-mers in a string of X have indeed a strong intrinsic relationship in that they share an overlap of k−1 symbols. It seems profitable to exploit the overlap information to preserve (as much as possible) the local relationship between consecutive *k*-mers as to reduce the randomness of *f*, thus lowering its bit complexity and evaluation time.

In particular, we are interested in the design of a locality-preserving MPHF in the following sense. Given a query sequence *Q*, if f(x)=j for some *k*-mer x∈Q, we would like *f* to hash Next(x) to j+1, Next(Next(x)) to j+2, and so on, where Next(x) is the *k*-mer following *x* in *Q* (assuming Next(x) and Next(Next(x)) are in X as well). This behavior of *f* is very desirable in practice, at least for two important reasons. First, it implies compression for satellite values associated to *k*-mers. Typical satellite values are abundance counts, reference identifiers (sometimes called “colors”), or contig identifiers (e.g. unitigs) in a de Bruijn graph. Consecutive *k*-mers tend to have very similar—if not identical—satellite values, hence hashing consecutive *k*-mers to consecutive identifiers induce a natural clustering of the associated satellite values which is amenable to effective compression. The second important reason is, clearly, faster evaluation time when querying for consecutive *k*-mers in a sequence. This streaming query modality is the query modality employed by *k*-mer-based applications (Almodaresi et al. 2018; Bingmann et al. 2019; Marchet et al. 2021; Robidou and Peterlongo 2021; Pibiri 2022b).

We formalize the notion of locality-preserving MPHF along with other preliminary definitions in Section 2. We show how to obtain a locality-preserving MPHF in very compact space in Section 3. To achieve this result, we make use of two algorithmic tools: random minimizers (Schleimer et al. 2003; Roberts et al. 2004) and a novel partitioning scheme for sub-sequences of consecutive *k*-mers sharing the same minimizers (super-*k*-mers) which allows a more parsimonious memory layout. The space of the proposed solution decreases for growing *k* and the data structure is built in linear time in the size of the input (number of distinct *k*-mers). In Section 4 we present experiments across a breadth of datasets to show that the construction is practical too: the functions can be several times smaller and even faster to query than the most efficient, albeit “general-purpose”, MPHFs. We conclude in Section 5 where we also sketch some promising future directions. Our C++ implementation of the method is publicly available at https://github.com/jermp/lphash.

## 2 Notation and definitions

Let X be a set of strings over an alphabet Σ. Throughout the article, we focus on the DNA alphabet Σ={A,C,G,T} to better highlight the connection with our concrete application but our algorithms can be generalized to work for arbitrary alphabets. A sub-string of length *k* of a string S∈X is called a *k*-mer of *S*.

Definition 1 (Spectrum). *The k-mer spectrum of* X*is the set of all distinct k-mers of the strings in* X*. Formally:* ${\text{spectrum}}_{k}(X):=\{x\in \Sigma^{k}|\exists S\in X\;\text{such that}\;x\;\text{is}\;a\;k-\mathrm{mer}\;\text{of}\;S\}.$

Definition 2 (Spectrum-Preserving String Set). *A spectrum-preserving string set (or SPSS)* S*of* X*is a set of strings such that (i) each string of* S*has length at least k, and (ii)* ${\text{spectrum}}_{k}(S)={\text{spectrum}}_{k}(X)$.

Since our goal is to build a MPHF for the *k*-mers in a SPSS, we are interested in a SPSS S where each *k*-mer is seen only once, i.e. for each *k*-mer $x\in {\text{spectrum}}_{k}(S)$ there is only one string of S where *x* appears once. We assume that no *k*-mer appearing at the end of a string shares an overlap of k−1 symbols with the first *k*-mer of another string, otherwise we could reduce the number of strings in S and obtain a smaller SPSS. In the following, we make use of this form of SPSS which is suitable for the minimal perfect hashing problem. We remark that efficient algorithms exist to compute such SPSSs (see e.g. Rahman and Medvedev 2020; Břinda et al. 2021; Khan and Patro 2021; Khan et al. 2022).

The input for our problem is therefore a SPSS S for X with |S| strings and n>1 distinct *k*-mers. Without loss of generality, we index *k*-mers based on their positions in S, assuming an order $S_{1},S_{2},S_{3},\ldots$ of the strings of S is fixed, and we indicate with $x_{i}$ the *i*-th *k*-mer in S, for i=1,…,n.

We want to build a MPHF $f:\Sigma^{k}\to [n]$ for S; more precisely, for the *n* distinct *k*-mers in ${\text{spectrum}}_{k}(S)$. We remark again that our objective is to exploit the overlap of k−1 symbols between consecutive *k*-mers from a string of S to preserve their locality, and hence reduce the bit complexity of *f* as well as its evaluation time when querying *k*-mers in sequence.

We define a locality-preserving MPHF, or LP-MPHF, for S as follows.

Definition 3 (LP-MPHF). *Let* $f:\Sigma^{k}\to [n]$*be a MPHF for*S*and A be the set* $\left\{1\leq i<n|\exists S\in S,x_{i},x_{i+1}\in S\wedge f(x_{i+1})=f(x_{i})+1\right\}$*. The function f is* (1−ε)*-locality-preserving for* S*if* ε≥1−|A|/n.

Intuitively, the “best” LP-MPHF for S is the one having the smallest ε, so we look for practical constructions with small ε. On the other hand, note that a “classic” MPHF corresponds to the case where the locality-preserving property is almost always not satisfied and, as a consequence, ε will be ∼1.

Two more considerations are in order. First, it should be clear that the way we define locality-preservation in Definition 3 is only pertinent to SPSSs where having consecutive hash codes for consecutive *k*-mers is a very desirable property as motivated in Section 1. A different definition of locality-preservation could instead be given if we were considering generic input keys. Second, we did not use the term “order-preserving” to stress the distinction from classic order-preserving functions in the literature (Fox et al. 1991) that make it possible to preserve any wanted order and, as such, incur in an avoidable Ω(log n)-bit overhead per key. Here, we are interested in preserving only the input order of the *k*-mers which is the one that matters in practice.

Definition 4 (Fragmentation Factor). *Given a SPSS* S*with* |S|*strings and* $n=|{\text{spectrum}}_{k}(S)|$*distinct k-mers, we define the fragmentation factor of* S*as* α:=(|S|−1)/n.

The fragmentation factor of S is a measure of how contiguous the *k*-mers in S are. The minimum fragmentation α=0 is achieved for |S|=1 and, in this case, $x_{i}$ shares an overlap of k−1 symbols with $x_{i+1}$ for “all” i=1,…,n−1. This ideal scenario is, however, unlikely to happen in practice. On the other hand, the worst-case scenario of maximum fragmentation α=1−1/n is achieved when |S|=n and *k*-mers do not share any overlap (of length k−1). This is also unlikely to happen given that *k*-mers are extracted consecutively from the strings of X and, as a result, many overlaps are expected. A more realistic scenario happens, instead, when |S|≪n, resulting in ε≫α. For the rest of the paper, we focus on this latter scenario to make our analysis meaningful.

From Definitions 3 and 4, it is easy to see that ε≥1/n when α=0, and ε=1 when α=1−1/n. In general, we have ε≥α+1/n since there are at least |S|−1 indexes *i* for which $f(x_{i+1})\neq f(x_{i})+1$. How small ε can actually be therefore depends on the input SPSS (and on the strategy used to implement *f* in practice, as we are going to illustrate in Section 3).

Lastly in this section, we define minimizers and super-*k*-mers that will be one of the main ingredients used in Section 3.

Definition 5 (Random Minimizer of a *k*-mer). *Given a k-mer x and a random hash function h, the minimizer of x is any m-mer* μ*such that* h(μ)≤h(y)*for any other m-mer y of x, for some* m≤k.

In case the minimizer of *x* is not unique, we break ties by taking the leftmost *m*-mer in *x*. For convenience, we indicate with w=k−m+1 the number of *m*-mers in a *k*-mer. (Note that Definition 5 defines a minimizer as a specific *m*-mer inside a *k*-mer rather than a specific *k*-mer in a window of *w* consecutive *k*-mers, which is the more standard definition found in the literature.) Since *h* is a random hash function (with a wide range, e.g. $\left[1..2^{64}\right]$), each *m*-mer in a *k*-mer has probability $\approx \frac{1}{w}$ of being the minimizer of the *k*-mer. We say that the triple (k,m,h) defines a random minimizer scheme. The density of a minimizer scheme is the expected number of selected minimizers from the input.

Definition 6 (Super-*k*-mer). *Given a string S, a super-k-mer g is a maximal sub-string of S where each k-mer has the same minimizer* μ*and* μ*appears only once in g.*

## 3 Locality-preserving minimal perfect hashing of *k*-mers

In this section, we describe an algorithm to obtain locality-preserving MPHFs for a spectrum-preserving string set S. The algorithm builds upon the following main insight.

**Figure 1.**
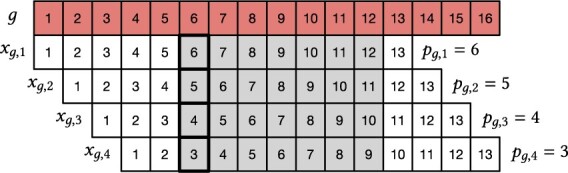
A super-*k*-mer *g* of length 16 with |g|−k+1=16−13+1=4*k*-mers $x_{g,1},x_{g,2},x_{g,3},x_{g,4}$ for k=13 and minimizer length m=7. The shaded boxes highlight the minimizer whose start position is $p_{g,i}$ in *k*-mer $x_{g,i}$. It is easy to see that $i=p_{g,1}-p_{g,i}+1$ for any 1≤i≤|g|−k+1.

**Implicitly ranking *k*-mers through minimizers.** Let *g* be a super-*k*-mer of some string S∈S and assume *g* is the only super-*k*-mer whose minimizer is μ. By definition of super-*k*-mer, all the *k*-mers $x_{g,1},\ldots ,x_{g,|g|-k+1}$ in *g* contain the minimizer μ as a sub-string—$x_{g,i}$ being the *i*-th *k*-mer of *g*. If $p_{g,1}$ is the start position of μ in the first *k*-mer $x_{g,1}$ of *g*, then is the start position of μ in $x_{g,i}$ for 1≤i≤|g|−k+1. Figure 1 gives a practical example for a super-*k*-mer *g* of length 16 and k=13.


(1) $$p_{g,i}=p_{g,1}-i+1,$$

The next property illustrates the relation between the size |g|−k+1 of the super-*k*-mer *g* and the position $p_{g,1}$ (we will come later on the implications of this property).

Property 1. $|g|-k+1\leq p_{g,1}\leq w$*for any super-k-mer g.*

**Proof.** Since $p_{g,1}$ is the start position of the minimizer in the first *k*-mer of *g*, there are at most $p_{g,1}$*k*-mers that contain the minimizer as a sub-string, hence $|g|-k+1\leq p_{g,1}$. However, *g* cannot contain more than *w k*-mers. □

Now, suppose we are given a query *k*-mer x∈S whose minimizer is μ. The *k*-mer must appear as a sub-string of *g*, i.e. it must be one among $x_{g,1},\ldots ,x_{g,|g|-k+1}$. We want to compute the rank of *x* among the *k*-mers $x_{g,1},\ldots ,x_{g,|g|-k+1}$ of *g*, which we indicate by Rank(x) (assuming that it is clear from the context that Rank is relative to *g*). Let *p* be the start position of μ in *x*. We can use this positional information *p* to compute Rank(x) as follows:

- if $p_{g,1}\geq p$ and $1\leq p_{g,1}-p+1\leq |g|-k+1$, then
  (2) $$\text{Rank}(x)=p_{g,1}-p+1$$
- otherwise ($p_{g,1}<p$ or $p_{g,1}-p+1>|g|-k+1$), *x* cannot possibly be in *g* and, hence, indexed by *f*.

Our strategy is to compute $f(x_{g,i})$ as for any *k*-mer $x_{g,1},\ldots ,x_{g,|g|-k+1}$ of *g*. Next, we show in Lemma 1 that this strategy maps the *k*-mers $x_{g,1},\ldots ,x_{g,|g|-k+1}$ bijectively in $\left\{(f(x_{g,1})-1)+1,\ldots ,(f(x_{g,1})-1)+|g|-k+1\right\}$*and preserves their locality* (i.e. their relative order in *g*).

Lemma 1. *The strategy in Equation (3) guarantees*$f(x_{g,i+1})=f(x_{g,i})+1$*for any* i=1,…,|g|−k.

(3) $$f(x_{g,i})=f(x_{g,1})+\text{Rank}(x_{g,i})-1=f(x_{g,1})+p_{g,1}-p_{g,i},$$

**Proof.** For Equation (3), $f(x_{g,i})=f(x_{g,1})+p_{g,1}-p_{g,i}$. Therefore, $f(x_{g,i+1})=f(x_{g,1})+p_{g,1}-p_{g,i+1}$. Since $p_{g,i+1}=p_{g,i}-1$ for Equation (1), then $f(x_{g,i+1})=f(x_{g,1})+p_{g,1}-p_{g,i+1}=f(x_{g,1})+p_{g,1}-p_{g,i}+1=f(x_{g,i})+1$. □

To sum up, the position of the minimizer in the first *k*-mer of *g*, $p_{g,1}$, defines an implicit ranking (i.e. achieved without explicit string comparison) of the *k*-mers inside a super-*k*-mer.

### 3.1 Basic data structure

From Equation (3) is evident that $f(x_{g,1})$ acts as a “global” component in the calculation of $f(x_{g,i})$, which must be added to a “local” component represented by $\text{Rank}(x_{g,i})$. We have already shown how to compute $\text{Rank}(x_{g,i})$ in Equation (2): Lemma 1 guarantees that this local rank computation bijectively maps the *k*-mers of *g* into [1..|g|−k+1]. We are therefore left to show how to compute $f(x_{g,1})$ for each super-*k*-mer *g*. We proceed as follows.

Algorithm 1. Evaluation algorithm for *f*, given the *k*-mer *x*. The helper function minimizer(x) computes the minimizer μ of *x* and the starting position *p* of μ in *x*.

1: **function** *f*(*x*):

2:  (μ,p)=minimizer(x)

3:  $i=f_{m}(\mu )$

4:  **return**L[i]+P[i]−p

**Layout.** Let M be the set of all the distinct minimizers of S. We build a MPHF for M, $f_{m}:\Sigma^{m}\to [|M|]$. Assume, for ease of exposition, that each super-*k*-mer *g* is the only super-*k*-mer having minimizer μ. (We explain how to handle the case where more super-*k*-mers have the same minimizer in Section 3.3.) We allocate an array $L^{\prime }[1..|M|+1]$ where $L^{\prime }[1]=0$ and $L^{\prime }[f_{m}(\mu )+1]=|g|-k+1$ for every minimizer μ. We then take the prefix-sums of $L^{\prime }$ into another array *L*, that is, $L[i]=\sum_{j=1}^{i}L^{\prime }[j]$ for all i=2,…,|M|+1. We therefore have that $L[f_{m}(\mu )]$ indicates the number of *k*-mers before those in *g* (whose minimizer is μ) in the order given by $f_{m}$. The size of *g* can be recovered as $L[f_{m}(\mu )+1]-L[f_{m}(\mu )]=|g|-k+1$. In conclusion, we compute $f(x_{g,1})$ as $L[f_{m}(\mu )]$. The positions $p_{1}$ of each super-*k*-mer *g* are instead written in another array P[1..|M|] where $P[f_{m}(\mu )]=p_{1}$.

It follows that the data structure is built in O(n) time, since a scan over the input suffices to compute all super-*k*-mers and $f_{m}$ can be built in O(|M|) expected time.

**Lookup.** With these three components—$f_{m}$, and the two arrays *L* and *P—*it is easy to evaluate f(x) as shown in Algorithm 1. The complexity of the lookup algorithm is O(w) since this is the complexity of computing the minimizer (assuming each hash calculation to take constant time) and the overall evaluation of $f_{m}$ as well, since accessing the arrays *L* and *P* takes O(1).

**Compression.** The data structure for *f* itself is a compressed representation for $f_{m}$, *L*, and *P*. To compute the space taken by the data structure we first need to know |M|—the expected number of distinct minimizers seen in the input. Assuming again that there are no duplicate minimizers, if *d* indicates the density of a random minimizer scheme, then

-
|M|=dn , and
-
ε=d as a direct consequence of Lemma 1.

In particular, a result due to Zheng et al. (2020, Theorem 3) allows us to compute *d* for a random minimizer scheme as $d=\frac{2}{w+1}+o(1/w)$ if $m>(3+\epsilon ){\;\text{log}\;}_{4}(w+1)$ for any ϵ>0. We will always operate under the condition that *m* is sufficiently large compared to *k* otherwise minimizers are meaningless.

Therefore, any random minimizer scheme gives us a (1−ε)-LP MPHF with $\varepsilon =\frac{2}{w+1}$ (we omit lower order terms for simplicity) as illustrated in the following theorem (see the Supplementary Material for the proof).

Theorem 1. *Given a random minimizer scheme* (k,m,h)*with* $m>(3+\epsilon ){\;\text{log}\;}_{4}(w+1)$*for any* ϵ>0*and* w=k−m+1*, there exists a* (1−ε)*-LP MPHF for a SPSS* S*with* $n=|{\text{spectrum}}_{k}(S)|$*which takeswhere* $\varepsilon =\frac{2}{w+1}$*and b is a constant larger than* ${\;\text{log}\;}_{2}(e)$.

$$n\cdot \frac{2}{w+1}({\;\text{log}\;}_{2}(4{\left(w+1\right)}^{2})+b+o(1))\;\text{bits}$$

Note that the space bound in Theorem 1 decreases as *w* grows; for example, when *m* is fixed and *k* grows. Next we show how to improve this result using some structural properties of super-*k*-mers.

### 3.2 Partitioned data structure

Property 1 states that $|g|-k+1\leq p_{g,1}\leq w$ for any super-*k*-mer *g*. As an immediate implication we have that if |g|−k+1=w then also $p_{g,1}=w$ (and, symmetrically, if $p_{g,1}=1$ then |g|=k). This suggests that, whenever a super-*k*-mer contains a maximal number of *k*-mers, then we can always implicitly derive that $|g|-k+1=p_{g,1}=w$. We can thus save the space for the entries dedicated to such super-*k*-mers in the arrays *L* and *P*. Note that the converse is not true in general, i.e. if $p_{g,1}=w$ it could be that |g|−k+1<w. Nonetheless, we can still save space for some entries of *P* in this case.

Depending on the starting position of the minimizer in the first and last *k*-mer of a super-*k*-mer, we distinguish between four “types” of super-*k*-mers (Definition 7).

Definition 7 (FL rule). *Let g be a super-k-mer. The first/last (FL) rule is as follows:*

- *if* $p_{g,1}=w$*and* $p_{g,|g|-k+1}=1$*, then g is left-right-max; else*
- *if* $p_{g,1}<w$*and* $p_{g,|g|-k+1}=1$*, then g is left-max; else*
- *if* $p_{g,1}=w$*and* $p_{g,|g|-k+1}>1$*, then g is right-max; else*
- *if* $p_{g,1}<w$*and* $p_{g,|g|-k+1}>1$*, then g is non-max.*

**Figure 2.**
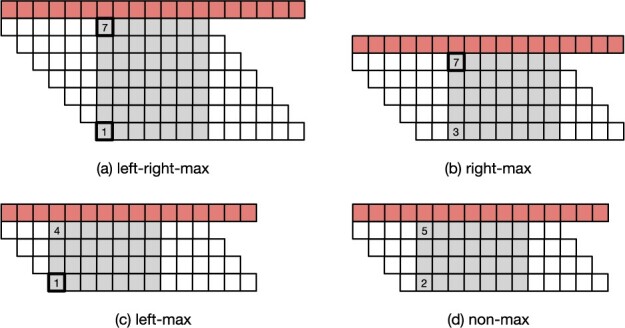
The four different types of super-*k*-mers. The example is for k=13 and minimizer length m=7, so w=k−m+1=13−7+1=7. The shaded boxes highlight the minimizer sub-string inside a *k*-mer. The start position of the minimizer is marked with a solid border when it is either max (7), or min (1).

See Fig. 2 for a schematic illustration.

**Layout.** Based on the FL-rule above, we derive a partitioned layout as follows. We store the type of each super-*k*-mer in an array R[1..|M|], in the order given by $f_{m}$. We can now exploit this labeling of super-*k*-mers to improve the space bound of Theorem 1 because:

- for all left-right-max super-*k*-mers, we do not store *L* nor *P*;
- for all left/right-max super-*k*-mers, we only store *L—*precisely, two arrays $L_{l}$ and $L_{r}$ for left- and right-max super-*k*-mers, respectively;
- for all the other super-*k*-mers, i.e. non-max, we store both *L* and *P* as explained before—let us indicate them with $L_{n}$ and $P_{n}$ in the following.

Addressing the arrays $L_{l}$, $L_{r}$, $L_{n}$ and $P_{n}$, can be achieved by answering ${\text{Rank}}_{t}(i)$ queries on *R*: the result of this query is the number of super-*k*-mers that have type *t* in the prefix *R[*1.*i]*. If $i=f_{m}(\mu )$, then we read the type of the super-*k*-mer associated to μ as t=R[i]. Then we compute $j={\text{Rank}}_{t}(i)$. Depending on the type *t*, we either do not perform any array access or access the *j*-th position of either $L_{l}$, or $L_{r}$, or $L_{n}$ and $P_{n}$ (see Algorithm 2).

**Figure 3.**
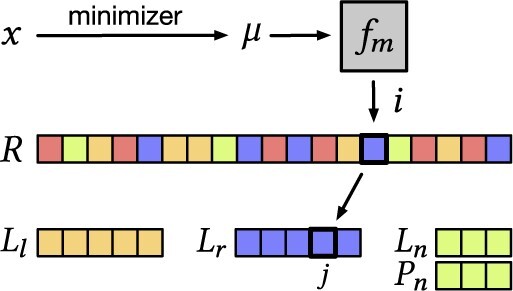
Partitioned data structure layout and the flow of Algorithm 2 for a query *k*-mer *x*, whose minimizer is μ, and with $i=f_{m}(\mu )$. Different colors in *R* are used to distinguish between the different super-*k*-mer types.

A succinct representation of *R* that also supports ${\text{Rank}}_{t}(i)$ and Access(i) queries is the wavelet tree (Grossi et al. 2003). In our case, we only have four possible types, hence a 2-bit integer is sufficient to encode a type. The wavelet tree therefore represents *R* in 2|M|+o(|M|) bits [The o(|M|) term is the redundancy needed to accelerate the binary rank queries. In practice, the term o(|M|) can be non-negligible, e.g. can be as high as 2⋅(|M|/4) bits using the Rank9 index (Vigna 2008, Section 3), but it is necessary for fast queries in practice (namely, O(1) time). Looking at Table 1a from Pibiri and Kanda (2021), we see that the redundancy is in between 3% and 25% of 2|M|.] and supports both queries in O(1) time. The wavelet tree is also built in linear time, so the building time of the overall data structure remains O(n). Refer to Fig. 3 for a pictorial representation of this partitioned layout.

**Table 1.** Computed (compt.) probabilities with Theorem 2 versus measured (measr.) using the whole human genome for three representative (k,m) configurations.

	k=31,m=21		k=47,m=26		k=63,m=28	
	compt.	measr.	compt.	measr.	compt.	measr.
$P_{lr}$	0.297	0.281	0.273	0.264	0.264	0.257
$P_{l}$	0.248	0.261	0.249	0.256	0.250	0.254
$P_{r}$	0.248	0.261	0.249	0.256	0.250	0.254
$P_{n}$	0.207	0.197	0.228	0.224	0.236	0.235

**Lookup.** Algorithm 2 gives the lookup algorithm for the partitioned representation of *f*. The complexity of the algorithm is still O(w) like that of the un-partitioned counterpart, Algorithm 1. The evaluation algorithm must now distinguish between the four different types of minimizer. On the one hand, this distinction involves an extra array access (to *R*) and a rank query as explained above but, on the other hand, it permits to save 2 array accesses in the left-right-max case or 1 in the left/right-max case compared to Algorithm 1 that always performs 2 array accesses (one access to *L* and one to *P*). Hence, the overall number of array accesses performed by Algorithm 2 is on average the same as that of Algorithm 1 assuming the four cases are equally likely (see next paragraph). For this reason we do not expect Algorithm 2 to incur in a penalty at query time compared to Algorithm 1 despite of its more complex evaluation.

Algorithm 2. Evaluation algorithm for a partitioned representation of *f*. The quantities $n_{lr}$, $n_{l}$, $n_{r}$, and $n_{n}$ are, respectively, the number of left-right-max, left-max, right-max, and non-max super-*k*-mers of S.

1: **function** *f*(*x*):

2:  (μ,p)=minimizer(x)

3:  $i=f_{m}(\mu )$

4:  t=R[i]

5:  $j={\text{Rank}}_{t}(i)$

6:  prefix=0, offset=0, $p_{1}=0$

7:  **switch**(*t*):

8:    **case** left-right-max:

9:      prefix=0, offset=(j−1)w, $p_{1}=w$

10:    **break**

11:   **case** left-max:

12:    $\mathrm{prefix}=n_{lr}$, $\mathrm{offset}=L_{l}[j]$, $p_{1}=L_{l}[j+1]-L_{l}[j]$

13:    **break**

14:   **case** right-max:

15:    $\mathrm{prefix}=n_{lr}+n_{l}$, $\mathrm{offset}=L_{r}[j]$, $p_{1}=w$

16:    **break**

17:   **case** non-max:

18:    $\mathrm{prefix}=n_{lr}+n_{l}+n_{r}$, $\mathrm{offset}=L_{n}[j]$, $p_{1}=P_{n}[j]$

19:    **break**

20:  **return**$\mathrm{prefix}+\mathrm{offset}+p_{1}-p$

**Compression.** Intuitively, if the fraction of left-right-max super-*k*-mers and that of left/right-max super-*k*-mers is sufficiently high, we can save significant space compared to the data structure in Section 3.1 that stores both *L* and *P* for all minimizers. We therefore need to compute the proportions of the different types of super-*k*-mers as given by the FL rule. For ease of notation, let $P_{lr}=P[g\;\text{is}\;\text{left}-\text{right}-\text{max}]$, $P_{l}=P[g\;\text{is}\;\text{left}-\text{max}]$, $P_{r}=P[g\;\text{is}\;\text{right}-\text{max}]$, $P_{n}=P[g\;\text{is}\;\text{non}-\text{max}]$, for any super-*k*-mer *g*.

Remark 1. *The FL rule is a partitioning rule, i.e.* $P_{lr}+P_{l}+P_{r}+P_{n}=1$*for any super-k-mer.*

Our objective is to derive the expression for the probabilities $P_{lr}$, $P_{l}$, $P_{r}$, and $P_{n}$, parametric in *k* (*k*-mer length) and *m* (minimizer length). To achieve this goal we propose a simple model based on a (discrete-time) Markov chain.

**Figure 4.**
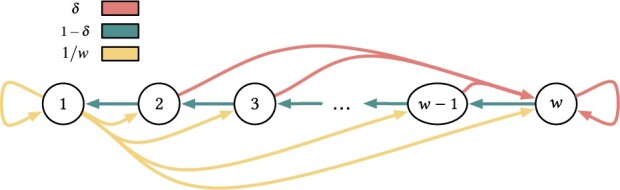
The chain is in state 1≤p≤w if the minimizer starts at position *p* in the *k*-mer. Different edge colors represent different probabilities.

Let $X:\Sigma^{k}\to \{1,\ldots ,w\}$ be a discrete random variable, modeling the starting position of the minimizer in a *k*-mer. The corresponding Markov chain is illustrated in Fig. 4. Each state of the chain is labeled with the corresponding value assumed by *X*, i.e. with each value in {1,…,w}. Clearly, we have a left-right-max super-*k*-mer if, from state *w* we transition to state w−1, then to w−2, …, down to state 1. Each state has a fallback probability to go to state *w* which corresponds to the event that the right-most *m*-mer (that coming next to the right) is the new minimizer. If the chain reaches state 1, instead, we know that we are always going to see a new minimizer next. If c∈[1..u] is the code assigned to the current minimizer by the coding function *h* used by μ, for some universe size *u* (e.g. if *c* is a 64-bit hash code, then $u=2^{64}$), the probability for any *m*-mer to become the new minimizer is equal to $\delta =\frac{c-1}{u}$. Vice versa, the probability of keeping the same minimizer when sliding one position to the right, is 1−δ. Whenever we change minimizer, we generate a new code *c* and, hence, the probability δ changes with every formed super-*k*-mer. Nonetheless, the following Theorem shows that the probabilities $P_{lr}$, $P_{l}$, $P_{r}$, and $P_{n}$, do not depend on δ.

Theorem 2. *For any random minimizer scheme* (k,m,h)*we have*

$$\begin{array}{c} P_{lr}=P[g\;\text{is}\;\text{left}-\text{right}-\text{max}]=W^{2}+1/w\\ P_{l}=P[g\;\text{is}\;\text{left}-\text{max}]=W(1-W)\\ P_{r}=P[g\;\text{is}\;\text{right}-\text{max}]=W(1-W)\\ P_{n}=P[g\;\text{is}\;\text{non}-\text{max}]=W^{2} \end{array}$$

*where* $W=\frac{1}{2}\cdot (1-\frac{1}{w})$*and* w=k−m+1.

We give the following lemma to prove Theorem 2. (When we write “first”/“last” *k*-mer we are going to silently assume “of a super-*k*-mer”.)

Lemma 2. $P[X=1]=\frac{1}{2}$*and* $P[X=w]=\frac{1}{2}\cdot (1+\frac{1}{w})$.

**Proof.** First note that for any 1≤p≤w−1. Then we have the following equivalences.


(4) $$P[X=p\;\text{in}\;\text{the}\;\mathrm{first}\;k-\mathrm{mer}]=P[X=1]\cdot \frac{1}{w},$$

(5) $$\begin{array}{l} \sum_{p=1}^{w}P[X=p\;\text{in}\;\text{the}\;\mathrm{first}\;k-\mathrm{mer}]=1\Leftrightarrow \\ P[X=w]+\sum_{p=1}^{w-1}P[X=p\;\text{in}\;\text{the}\;\mathrm{first}\;k-\mathrm{mer}]=1\Leftrightarrow \\ P[X=w]+P[X=1]\cdot (1-\frac{1}{w})=1\;[\;\text{for}\;\text{Equation}\;4]. \end{array}$$

Now note that because the starting position of the minimizer of the **first** *k*-mer of any left-right-max and of any right-max super-*k*-mer is *w*. In a similar way, we have that because the starting position of the minimizer of the **last** *k*-mer of any left-right-max and of any left-max super-*k*-mer is 1. Now note that $P_{l}=P_{r}$ because:


(6) $$P[X=w]=P_{lr}+P_{r}$$

(7) $$\begin{array}{l} P[X=1\;\text{in}\;\text{the}\;\mathrm{last}\;k-\mathrm{mer}]=\\ P[X=1]+P[X=1\;\text{in}\;\text{the}\;\mathrm{first}\;k-\mathrm{mer}]=\\ P[X=1]\cdot (1+\frac{1}{w})\;[\text{for}\;\text{Equation}\;4\;\text{with}\;p=1]\\ =P_{lr}+P_{l} \end{array}$$

$$\begin{array}{l} P_{l}=P[X=w\;\text{in}\;\mathrm{first}\;k-\mathrm{mer}]\cdot P[X\neq 1\;\text{in}\;\mathrm{last}\;k-\mathrm{mer}]=\\ =(1-P[X\neq w\;\text{in}\;\mathrm{first}\;k-\mathrm{mer}])\cdot (1-P[X=1\;\text{in}\;\mathrm{last}\;k-\mathrm{mer}])=\\ =(1-P[X=1]\cdot (1-\frac{1}{w}))\cdot (1-P[X=1]\cdot (1+\frac{1}{w}))=\\ ={\left(P[X=1]\right)}^{2}\cdot (1-\frac{1}{w^{2}}),\;\text{and}\;\text{similarly}\\ P_{r}=P[X\neq w\;\text{in}\;\mathrm{first}\;k-\mathrm{mer}]\cdot P[X=1\;\text{in}\;\mathrm{last}\;k-\mathrm{mer}]=\\ =P[X=1]\cdot (1-\frac{1}{w})\cdot P[X=1]\cdot (1+\frac{1}{w})=\\ ={\left(P[X=1]\right)}^{2}\cdot (1-\frac{1}{w^{2}}). \end{array}$$

From equation $P_{l}=P_{r}$, we have $P_{lr}+P_{l}=P_{lr}+P_{r}$ which, using Equations (6) and (7), yields $P[X=w]=P[X=1]\cdot (1+\frac{1}{w})$. The Lemma follows by using the latter equation into Equation (5). □

Now we prove Theorem 2.

**Proof.** Since the FL rule induces a partition:


(8) $$\begin{array}{l} P_{lr}+P_{r}+P_{l}+P_{n}=1\Leftrightarrow \\ P_{lr}+P_{lr}+P_{r}+P_{l}+P_{n}=1+P_{lr}\\ {}[\text{adding}\;P_{lr}\;\text{to}\;\text{both}\;\text{sides}]\Leftrightarrow \\ 2P[X=w]+P_{n}=1+P_{lr}\\ {}[\text{knowing}\;\text{that}\;P_{lr}+P_{r}=P_{lr}+P_{l}=P[X=w]]\Leftrightarrow \\ P_{lr}=P_{n}+\frac{1}{w}[\text{for}\;\text{Lemma}\;2]. \end{array}$$

Again exploiting the fact that $P_{lr}+P_{r}=P_{lr}+P_{l}=P[X=w]$, we also have


(9) $$P_{l}=P_{r}=P[X=w]-P_{lr}=\frac{1}{2}\cdot (1+\frac{1}{w})-P_{n}-\frac{1}{w}.$$

We have therefore to compute $P_{n}$ to also determine $P_{lr}$, $P_{l}$, and $P_{r}$.


(10) $$\begin{array}{l} P_{n}=P[X\neq w\;\text{in}\;\mathrm{first}\;k-\mathrm{mer}]\cdot P[X\neq 1\;\text{in}\;\mathrm{last}\;k-\mathrm{mer}]=\\ P[X=1]\cdot (1-\frac{1}{w})\cdot (1-P[X=1\;\text{in}\;\mathrm{last}\;k-\mathrm{mer}])=\\ P[X=1]\cdot (1-\frac{1}{w})\cdot (1-P[X=1]\cdot (1+\frac{1}{w}))=\\ {\left(\frac{1}{2}\cdot (1-\frac{1}{w})\right)}^{2}[\;\text{for}\;\text{Lemma}\;2]. \end{array}$$

Now letting $W=\frac{1}{2}\cdot (1-\frac{1}{w})$ and substituting $P_{n}=W^{2}$ [Equation (10)] into Equations (8) and (9), the Theorem follows. □

In Table 1, we report the probabilities $P_{lr}$, $P_{l}$, $P_{r}$, and $P_{n}$ computed using Theorem 2 for some representative combinations of *k* and *m* (these combinations are some of those used in the experiments of Section 4; see also Table 2). For comparison, we also report the probabilities measured over the whole human genome. We see that the probabilities computed with the formulas in Theorem 2 accurately model the empirical probabilities.

**Table 2.** Minimizer length *m* by varying *k* on the different datasets.

	k→	31	35	39	43	47	51	55	59	63
Yeast		15	15	16	16	16	16	18	18	18
Elegans		16	18	18	20	20	20	20	20	20
Cod		20	20	22	22	22	24	24	24	24
Kestrel		20	20	22	22	22	24	24	24	24
Human		21	21	23	23	26	26	28	28	28

The net result is that, for sufficiently large *w*, the probabilities in Theorem 2 are all approximately equal to 1/4, so that we have $\approx \frac{n}{2(w+1)}$ super-*k*-mers of each type. This also implies that the choice of 2-bit codes for the symbols of *R* is essentially optimal. Under this condition, we give the following theorem (see the Supplementary Material for the proof).

Theorem 3. *Given a random minimizer scheme* (k,m,h)*with* $m>(3+\epsilon ){\;\text{log}\;}_{4}(w+1)$*for any* ϵ>0*and* w=k−m+1*, there exists a* (1−ε)*-LP MPHF for a SPSS* S*with* $n=|{\text{spectrum}}_{k}(S)|$*which takeswhere* $\varepsilon =\frac{2}{w+1}$*and b is a constant larger than* ${\;\text{log}\;}_{2}(e)$.

$$n\cdot \frac{2}{w+1}({\;\text{log}\;}_{2}(\frac{16\cdot 2^{1/4}}{3}(w+1))+b+o(1))\;\text{bits}$$

### 3.3 Ambiguous minimizers

Let $G_{\mu }$ be the set of super-*k*-mers whose minimizer is μ. The rank computation in Equation (2) can be used as long as $|G_{\mu }|=1$, i.e. whenever one single super-*k*-mer *g* has minimizer μ and, thus, the single $p_{g,1}$ unequivocally displace all the *k*-mers $x_{g,1},\ldots ,x_{g,|g|-k+1}$. When $|G_{\mu }|>1$ we say that the minimizer μ is ambiguous. It is a known fact that the number of such minimizers is very small for a sufficiently long minimizer length *m* (Chikhi et al. 2014; Jain et al. 2020; Pibiri 2022b), and the number decreases for growing *m*. For example, on the datasets used in Section 4, the fraction of ambiguous minimizers is in between 1% and 4%. However, they must be dealt with in some way.

Let ξ be the fraction of *k*-mers whose minimizers are ambiguous. Our strategy is to build a fallback MPHF for these *k*-mers. This function adds ξ⋅b bits/*k*-mer on top of the space of Theorem 1 and Theorem 3, where $b>{\;\text{log}\;}_{2}(e)$ is the number of bits per key spent by a MPHF of choice. The fallback MPHF makes our functions (1−ε+ξ)-locality-preserving.

To detect ambiguous minimizers, one obvious option would be to explicitly use an extra 1-bit code per minimizer. This would however result in a waste of 1 bit per minimizer for most of them since we expect to have a small percentage of ambiguous minimizers. To avoid these problems, we use the following trick. Suppose μ is an ambiguous minimizer. We initially pretend that μ is not ambiguous. For the unpartitioned data structure from Section 3.1, we set $L[f_{m}(\mu )]=0$. A super-*k*-mer of size 0 is clearly not possible, thus we use the value 0 to indicate that μ is actually ambiguous. We do the same for the partitioned data structure from Section 3.2: in this case, we set $L_{r}[f_{m}(\mu )]=0$ pretending the type of μ is right-max (but we could have also used the type left-max or non-max). To sum up, with just an extra check on the super-*k*-mer size we know if the query *k*-mer must be looked-up in the fallback MPHF or not.

We leave the exploration of alternative strategies to handle ambiguous minimizers to future work. For example, one can imagine a recursive data structure where, similarly to Shibuya et al. (2022), each level is an instance of the construction with different minimizer lengths: if level *i* has minimizer length $m_{i}$, then level i+1 is built with length $m_{i+1}>m_{i}$ over the *k*-mers whose minimizers are ambiguous at level *i*.

## 4 Experiments

In this section, we report on the experiments conducted to assess the practical performance of the method presented in Section 3, which we refer to as LPHash in the following. Our implementation of the method is written in C++ and available at https://github.com/jermp/lphash.

**Implementation details.** We report here the major implementation details for LPHash. The arrays *L* and *P* are compressed with Elias-Fano (Fano 1971; Elias 1974) to exploit its constant-time random access (see also Pibiri and Venturini 2021, Section 3.4) for an explanation of such compressed encoding). Both the function $f_{m}$ and the fallback MPHF are implemented with PTHash using parameters (D-D,α=0.94,c=3.0), unless otherwise specified. Under this configuration, the space taken by a PTHash MPHF is 2.3–2.5 bits/key.

We do not compress the bit-vectors in the wavelet tree and we add constant-time support for rank queries using the Rank9 index (Jacobson 1989; Vigna 2008). The Rank9 index adds 25% more space at each level of the wavelet tree, making the wavelet tree to take 2.5 bits per element in practice. Therefore, we estimate the little-Oh factor in Theorem 1 and Theorem 3 to be 0.5.

**Competitors.** We compare the space usage, query time, and building time of LPHash against PTHash (Pibiri and Trani a,b), the fastest MPHF in the literature, and the popular BBHash (Limasset et al. 2017). Both competitors are also written in C++. Following the recommendations of the respective authors, we tested two example configurations each:

- PTHash-v1, with parameters (D-D,α=0.94,c=5.0);
- PTHash-v2, with parameters (EF,α=0.99,c=5.0);
- BBHash-v1, with parameter γ=2;
- BBHash-v2, with parameter γ=1;

We point the reader to the respective papers for an explanation of such parameters; we just report that they offer a trade-off between space, query efficiency, and building time as also apparent in the following experiments.

**Testing machine.** The experiments were executed on a machine equipped with a Intel i9-9900K CPU (clocked at 3.60 GHz), 64 GB of RAM, and running the Linux 5.13.0 operating system. The whole code (LPHash and competitors) was compiled with gcc 11.2.0, using the flags -O3 and -march=native.

**Datasets.** We use datasets of increasing size in terms of number of distinct *k*-mers; namely, the whole-genomes of: *Saccharomyces cerevisiae* (Yeast, 11.6 × 10^6^*k*-mers), *Caenorhabditis elegans* (Elegans, 96.5 × 10^6^*k*-mers), *Gadus morhua* (Cod, 0.56 × 10^9^*k*-mers), *Falco tinnunculus* (Kestrel, 1.16 × 10^9^*k*-mers), and *Homo sapiens* (Human, 2.77 × 10^9^*k*-mers). For each dataset, we obtain the corresponding SPSS by first building the compacted de Bruijn graph using BCALM2 (Chikhi et al. 2016), then running the UST algorithm (Rahman and Medvedev 2020). At our code repository we provide detailed instructions on how to prepare the datasets for indexing. Also, all processed datasets are available at https://zenodo.org/record/7239205 already in processed form so that it is easy to reproduce our results.

### 4.1 Space effectiveness

To build an instance of LPHash for a given *k*, we have to choose a suitable value of minimizer length (*m*). A suitable value of *m* should clearly be not too small (otherwise, most minimizers will appear many times), nor too large (otherwise, the space of $f_{m}$ will be too large as well). In general, a good value for *m* can be chosen around ${\;\text{log}\;}_{4}(N)$ where *N* is the cumulative length of the strings in the input SPSS. Remember from our discussion in Section 3.3 that the fraction of ambiguous minimizers decreases for growing *m*. Therefore, testing LPHash for growing values of *k* allows us to progressively increase *m*, starting from $m={\;\text{log}\;}_{4}(N)$, while keeping w=k−m+1 sufficiently large and reducing the fraction of ambiguous minimizers as well. Following this principle, for each combination of *k* and dataset, we choose *m* as reported in Table 2.

**Figure 5.**
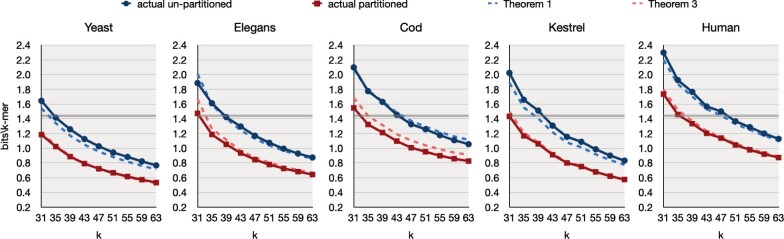
Space in average bits/*k*-mer for LPHash by varying *k*, for both unpartitioned and partitioned data structures. The flat solid line at ${\;\text{log}\;}_{2}(e)=1.442$ bits/*k*-mer indicates the classic MPHF lower-bound. Lastly, the dashed lines corresponds to the space bounds computed using Theorem 1 and Theorem 3 with b=2.5 and including the space for the fallback MPHF.

Figure 5 shows the space of LPHash in average bits/*k*-mer, by varying *k* from 31 to 63 with a step of 4, for both un-partitioned and partitioned data structures. We report the actual space usage achieved by the implementation against the space bounds computed using Theorem 1 (un-partitioned) and Theorem 3 (partitioned) for b=2.5. The *b* parameter models the number of bits per key spent by a MPHF of choice for the representation of the minimizer MPHF and the fallback MPHF. (For all datasets, we use c=3.0 for the PTHash $f_{m}$ and fallback, except on the largest Human where we use c=5.0 to lower construction time at the expense of a larger space usage.)

We make the following observations.

- The space bounds computed with Theorem 1 and Theorem 3 are very similar to the actual space usage of LPHash, thus confirming the correctness and accuracy of our analysis in Section 3.
- As expected, the space of LPHash lowers for increasing *k* and the partitioned data structure is always considerably smaller than the un-partitioned counterpart.
- We report the space taken by the tested competitive configurations in Table 3. Comparing the space values in Table 3 with those in Fig. 5, the net result is that the space of LPHash is much lower than that of the classic MPHFs traditionally used in the prior literature and in practice.

**Table 3.** Space in average bits/*k*-mer for PTHash and BBHash.^a^ As reference points, we also report the bits/k-mer for partitioned LPHash for three representative values of k (see also Fig. 5).

Method	*k*	Yeast	Elegans	Cod	Kestrel	Human
LPHash	31	1.18	1.47	1.55	1.43	1.74
	47	0.72	0.85	1.01	0.82	1.14
	63	0.53	0.64	0.83	0.58	0.87

PTHash-v1		2.76	2.68	2.65	2.58	2.65
PTHash-v2		2.20	2.13	2.09	2.06	2.04
BBHash-v1		3.71	3.71	3.71	3.71	3.71
BBHash-v2		3.06	3.06	3.06	3.06	3.06

The numbers reported in Table 3 were taken for *k* = 63 although the avg. bits/*k*-mer for PTHash and BBHash does not depend on *k*.

To make a concrete example, partitioned LPHash for k=63 achieves 0.53, 0.64, 0.83, 0.58, and 0.87 bits/*k*-mer on Yeast, Elegans, Cod, Kestrel, and Human respectively. These values are 5.1×, 4.1×, 3.2×, 4.4×, and 3× smaller than the those achieved by PTHash-v1 (and even smaller when compared to BBHash). Even compared to the most succinct configuration, PTHash-v2 (around 2 bits/*k*-mer), LPHash still retains 2.3–3.7× better space.

We remark that, however, PTHash and BBHash are “general-purpose” MPHFs that can work with arbitrary keys, whereas the applicability of LPHash is restricted to spectrum-preserving string sets.

### 4.2 Query time

Table 4 reports the query time for LPHash in comparison to PTHash and BBHash. Timings were collected using a single core of the processor. We query all *k*-mers read from the Human chromosome 13, for a total of $\approx 100\times 10^{6}$ queries. First of all, we report that query timings for un-partitioned and partitioned LPHash are the same, so we do not distinguish between the two data structures in Table 4. This meets our expectation regarding the average number of array accesses that the two query algorithms perform as explained in Section 3.2.

**Table 4.** Query time in average nanoseconds per *k*-mer.

Method	*k*	Yeast		Elegans		Cod		Kestrel		Human	
		stream	random	stream	random	stream	random	stream	random	stream	random
LPHash	31	29	110	40	118	79	144	84	145	107	162
	35	28	125	35	124	65	147	69	149	90	166
	39	27	130	32	131	60	149	63	153	82	166
	43	25	137	30	135	52	152	54	155	73	169
	47	24	145	28	143	47	155	49	159	69	172
	51	24	152	28	150	45	159	46	162	63	174
	55	23	157	26	157	41	165	42	167	59	176
	59	23	165	25	165	39	171	39	173	57	182
	63	22	174	24	172	37	180	37	179	53	188

PTHash-v1		24		46		67		72		72	
PTHash-v2		38		64		130		155		175	
BBHash-v1		42		118		170		175		175	
BBHash-v2		42		125		180		190		190	

We distinguish between streaming and random queries (lookups) for LPHash. Given a query string *Q*, we query for each *k*-mer read “consecutively” from *Q*, that is, for *Q[*1.*k]*, Q[2..k+1], Q[3..k+2], etc. We refer to the this query modality as streaming; anything else different from streaming is a random lookup (i.e. “random” here means “without locality”). LPHash is optimized for streaming lookup queries, whereas PTHash and BBHash do not benefit from any specific query order. In fact, the locality-preserving nature of LPHash makes the calculation of hashes for consecutive *k*-mers very cheap, as consecutive *k*-mers are likely to be part of the same super-*k*-mer.

Considering the result in Table 4, we see that LPHash’s streaming query time is in fact much smaller than random query time. Both timings are sensitive to the growth of *k*: while the streaming one slightly decreases for the better locality, the random one increases instead, for the more expensive hash calculations.

LPHash is as fast as PTHash-v1 (fastest configuration) for streaming queries on the smaller Yeast dataset, but actually up to 1.4–2× faster on the larger datasets Elegans, Cod, Kestrel, and Human. Instead, it is up to 4× faster than PTHash-v2. We stress that this is a remarkable result given that PTHash is the fastest MPHF in the literature, being 2–6× faster than other methods. Compared to BBHash, LPHash is 2× faster on Yeast and up to 4–5× faster on the larger datasets.

Random lookup time is, instead, slower for LPHash compared to PTHash: this is expected because the evaluation of LPHash is more complex (it involves computing the minimizer, accessing several arrays, and computing a rank using a wavelet tree). However, we do not regard this as a serious limitation since, as we already motivated, the streaming query modality is the one used in Bioinformatics tasks involving *k*-mers (Almodaresi et al. 2018; Bingmann et al. 2019; Marchet et al. 2021; Robidou and Peterlongo 2021; Pibiri 2022b). We also observe that the slowdown is more evident on the smaller datasets while it tends to diminish on the larger ones. Except for the smaller Yeast dataset, the random lookup time of LPHash is competitive with that of BBHash or better.

### 4.3 Building time

We now consider building time which is reported in Table 5. Both LPHash and PTHash were built limiting to 8GB the maximum amount of RAM to use before resorting to external memory. (There is no such capability in the BBHash implementation so BBHash took more RAM at building time than the other two constructions.)

**Table 5.** Total building time, including the time to read the input and serialize the data structure on disk. All constructions were run with four processing threads.

Method	Yeast	Elegans	Cod	Kestrel	Human
	mm:ss	mm:ss	mm:ss	mm:ss	mm:ss
LPHash	00:01	00:15	05:30	03:50	07:25
PTHash-v1	00:03	00:29	07:37	20:34	63:30
PTHash-v2	00:03	00:46	14:15	40:00	124:00
BBHash-v1	00:01	00:07	00:48	01:40	04:13
BBHash-v2	00:01	00:08	01:05	02:22	07:50

The building time for un-partitioned and partitioned LPHash is the same. LPHash is competitive with the fastest BBHash and significantly faster than PTHash on the larger datasets. Specifically, it is faster than PTHash over the entire set of *k*-mers since it builds two smaller PTHash functions ($f_{m}$ and fallback). The slowdown seen for Cod is due to the larger fallback MPHF, which is built with PTHash under a strict configuration (c=3.0) that privileges space effectiveness (and query efficiency) rather than building time. One could in principle use BBHash instead of PTHash for the fallback function, hence trading space for better building time. For example, recall that we use c=5.0 on Human for this reason.

## 5 Conclusion and future work

In this article, we initiate the study of locality-preserving MPHFs for *k*-mers. We propose a construction, named LPHash, that achieves very compact space by exploiting the fact that consecutive *k*-mers share overlaps of k−1 symbols. This allows LPHash to actually break the theoretical ${\;\text{log}\;}_{2}(e)$ bit/key barrier for MPHFs.

We show that a concrete implementation of the method is practical as well. Before this paper, one used to build a BBHash function over the *k*-mers and spend (approximately) 3 bits/*k*-mer and 100–200 nanoseconds per lookup. This work shows that it is possible to do significantly better than this when the *k*-mers come from a spectrum-preserving string set: for example, less than 0.6–0.9 bits/*k*-mer and 30–60 nanoseconds per lookup. Our code is open-source.

As future work, we plan to further engineer the current implementation to accelerate construction and streaming queries. Other strategies for sampling the strings could be used other than random minimizers (Frith et al. 2022); for example, the *Miniception* (Zheng et al. 2020) achieving $\varepsilon =\frac{1.67}{w}+o(1/w)$. Evaluating the impact of such different sampling schemes is a promising avenue for future research. Lastly, we also plan to investigate other strategies for handling the ambiguous minimizers. A better strategy is likely to lead to improved space effectiveness and faster construction.

## Supplementary Material

btad219_Supplementary_Data

## Data Availability

https://zenodo.org/record/7239205.

## Code availability

https://github.com/jermp/lphash.
