# Supplementary material for: Locality-preserving minimal perfect hashing of k-mers
Source: Bioinformatics. 2023 Jun 30;39(Suppl 1):i534–43. doi: 10.1093/bioinformatics/btad219 (PMC10311298; doi:10.1093/bioinformatics/btad219)
Supplement: btad219_Supplementary_Data [file btad219_supplementary_data.pdf]

OXFORD

Supplementary Material

Locality-Preserving Minimal Perfect Hashing of K-Mers

Giulio Ermanno Pibiri [1,2], Yoshihiro Shibuya [3], Antoine Limasset [4]

[1] Ca' Foscari University of Venice, Venice, Italy
[2] ISTI-CNR, Pisa, Italy
[3] University Gustave Eiffel, Marne-la-Vallée, France
[4] University of Lille and CNRS, Lille, France

Abstract

This document contains the Supplementary Material for the paper "Locality-Preserving Minimal Perfect Hashing of K-Mers".

Proof of Theorem 1

For Lemma 1 and the discussion Section 3.1, f can be implemented using the three components f_m, L, and P. The MPHF f_m can be realized using $2n/(w+1) \cdot b$ bits. The array L stores $2n/(w+1)$ integers whose sum is n since each element represents the number of k-mers in a super-k-mer. Therefore L can be represented with Elias-Fano [**??**] using at most $\frac{2n}{w+1} \cdot (\log_2(\frac{w+1}{2}) + 2 + o(1))$ bits. We can also represent P using this space bound. In fact, note that since $|g| - k + 1 \leq p_{g,1} \leq w$ for Property 1, we can store the quantities $(p_{g,1} - (|g| - k + 1))$ in P for any super-k-mer g. If we compute the prefix-sums for the array P, we can encoded it with Elias-Fano. The last element in the prefix-summed P is at most $\sum_g (w - (|g| - k + 1)) = w \cdot \frac{2n}{w+1} - n < 2n - n = n$. Summing these spaces, the claimed space bound follows. □

Proof of Theorem 3

We have to represent the following components: the minimizer MPHF f_m, the array R storing the types of the super-k-mers, and the arrays L_l, L_r, L_n, and P_n. The MPHF f_m takes (i) $2n/(w+1) \cdot b$ bits and R takes (ii) $2n/(w+1) \cdot (2 + o(1))$ bits. For the left-right-max super-k-mers we do not store anything and they cover $w \cdot \frac{n}{2(w+1)} \approx n/2$ of the total k-mers. The other half of the k-mers is handled by the other three super-k-mer types: we do not know the exact amount of k-mers per type; yet the space is highest when each partition takes the same amount of k-mers (uniform partitioning), i.e., $n/6$. The space for L_l plus that for L_r is then (iii) $2 \cdot \frac{n}{2(w+1)} \cdot (\log_2(n/6 \cdot 2(w+1)/n) + 2 + o(1)) = \frac{n}{w+1} \cdot (\log_2((w+1)/3) + 2 + o(1))$ bits. Similarly, the array L_n takes $\frac{n}{2(w+1)} \cdot (\log_2((w+1)/3) + 2 + o(1))$ bits. The space for the array P_n is instead $\frac{n}{2(w+1)} \cdot (\log_2(2(w+1)/3) + 2 + o(1))$ bits following a similar argument to that used in the proof of Theorem 1: the last element in the prefix-summed P_n is at most $\sum_{g \text{ is non-max}} (w - (|g| - k + 1)) = w \cdot \frac{n}{2(w+1)} - n/6 < n/2 - n/6 = n/3$. Therefore, the space for L_n plus that for P_n is (iv) $\frac{n}{w+1} \cdot (\log_2((w+1)/3) + 5/2 + o(1))$ bits. Summing spaces (i), (ii), (iii), (iv) together, the claimed space bound follows. □

© The Author 2022.
